# Experimental Parametric Model for Adhesion Wear Measurements in the Dry Turning of an AA2024 Alloy

**DOI:** 10.3390/ma11091598

**Published:** 2018-09-03

**Authors:** Moises Batista Ponce, Irene Del Sol Illana, Severo Raul Fernandez-Vidal, Jorge Salguero Gomez

**Affiliations:** Department of Mechanical Engineering & Industrial Design, Faculty of Engineering, University of Cadiz, Av. Universidad de Cadiz 10, E-11519 Puerto Real-Cadiz, Spain; irene.delsol@uca.es (I.D.S.I); raul.fernandez@uca.es (S.R.F.V.); jorge.salguero@uca.es (J.S.G.)

**Keywords:** cutting tool wear, secondary adhesion wear, turning, machining, aluminium

## Abstract

Adhesion wear is the main wear mechanism in the dry turning of aluminium alloys. This type of wear produces an adhesion of the machining material on the cutting tool, decreasing the final surface quality of the machining parts and making it more difficult to maintain industrial tolerances. This work studies the influence of the cutting parameters on the volume of material adhered to the cutting tool surface for dry machining of AA2024 (Al-Cu). For that purpose, a specific methodology based on the automatic image processing method that can obtain the area and the thickness of the adhered material has been designed. This methodology has been verified with the results obtained through 3D analysis techniques and compared with the adhered volume. The results provided experimental parametric models for this wear mechanism. These models are analytic approximations of experimental data. The feed rate mainly results in low cutting speed, while low depths of cut presents a different behaviour due to the low contact pressure. The unstable behaviour of aluminium adhesion on the cutting tool produces a high variability of results. This continuous change introduces variation in the process caused by the continuous change of the cutting tool geometry.

## 1. Introduction

The increase of cutting tool life is a highly attractive objective in industry for improving the profitability of industrial machining processes. Tool wear and control are a critical challenge in the machining process nowadays, although they have been addressed in classic studies [1,2,3,4].

Different approaches have been used for aluminium alloys in order to minimise this issue. On the one hand, coatings are proposed to improve the cutting tool life, with the most recommended one being diamond coatings [5,6], because they have a higher hardness like intermetallic particles [6]. However, they have refractory properties that increase the temperature of the cutting zone [7], making the machining of Al parts more difficult, especially when high cutting speeds (up to 600 m/min) cannot be achieved. Moreover, the use of diamond coatings increases the cost of the cutting tool and, in several cases, makes it unprofitable to the industry.

On the other hand, different lubricant solutions have been proposed in recent years, mainly based on the use of Minimum Quantity Lubrication (MQL) [8,9,10], high-pressure coolant [5,11] and cryogenic systems [12,13]. Nevertheless, due to its low environmental impact and associated costs, dry machining must also be considered for the machining of Al alloys [14,15], as it is the most sustainable solution for the turning process [8,16]. It does not involve pollution of air or water resources but is necessary for a cutting tool with low friction coefficient and adequate heat resistance [8].

However, dry machining of aluminium alloys is usually associated with high adhesion rates on the cutting tool surface. This wear mechanism is related to the increase of the friction coefficient [17] caused by the contact between the surfaces. This type of contact creates a rough surface and a low sliding contact between tool and work materials [18], increasing the contact pressure and particle transference from one surface to another [19]. This can be classified as primary—from the tool to the chip—or secondary, from the workpiece to the tool. In the latter case, the material can adhere to the cutting tool, giving rise to a Built-Up Edge (BUE), presenting adhesion in the closest areas of the cutting edge, and the Built-Up Layer (BUL), located in the rake face [19,20,21].

Traditionally, BUE formation is related with low cutting speed, while BUL is associated with high cutting speed due to the conventional behaviour of wear in different steel machining, as was studied by Krolczyk et al. [22] using a methodology for predicting the cutting tool life in stainless steel and its relation to surface roughness [23]. However, this behaviour change is dependent on the machined material.

BUL is also considered in turning of nickel alloys as one of the main causes of poor surface finishing, affecting residual stresses [24]. Cutting forces can also be affected, and some models would help to define the mechanistic of parameters modelling [25]. Cutting tool wear by adhesion mechanisms changes cutting forces and cutting temperature, affecting the quality of the machined workpieces. Nevertheless, only few works in the field of modelling address these aspects [26].

In the particular case of aluminium alloys, different authors [18,19,27,28,29,30] define the development of secondary adhesion wear in several steps:

- *Build of Primary BUL*. A layer of near-pure aluminium, with a very low quantity of copper, is adhered due to the thermomechanical conditions. Under high pressure and temperature, the matrix is softened and welded to the cutting tool rake face by pressure.
- *Build of BUE.* It is produced by the mechanical adhesion of the workpiece alloy close to the cutting edge.
- *Build of secondary BUL.* A mechanical extrusion of the workpiece material over the rake face takes place. The BUE and secondary BUL usually grow to a critical size. Once it is reached they are detached—at least partially—from the cutting tool, taking with them cutting tool particles, thereby damaging the tool.

This process is unstable and usually cyclic, being repeated several times during long-term tests. It is a dynamic wear mechanism in which successive chip material layers are welded and hardened on the surface, or are deposited on the cutting tool by thermic or mechanical actions. BUE changes continuously because the workpiece material is deposited and the tool particles are moved away. This phenomenon is usually related to the onset of an over cut, due to the added material that modifies the initial cutting edge geometry and, consequently, the initial cutting conditions [4]. In some cases, it can also be followed by a weakening of the cutting tool, causing in some cases a complete or catastrophic and unexpected fracture.

Trujillo et al. [27] explain that this complexity in the machining of aluminium, especially due to the dynamic behaviour, makes measuring adhesion more difficult. The measurement has to consider the modification of the initial cutting geometry, taking into account volume loss and volume increase, as proposed by Gomez-Parra et al. in [18]. There are several technologies to measure tool wear, such as Focus-Variation Microscopy (FMV) [31,32], SEM-3D or tomography [33], image pattern comparison [34], direct measurements based on optical methods [27,35] or intelligent prediction systems [36]. All of these methods have usually been implemented in wear control, and few of them have been implemented for aluminium machining [27,32,33]. Moreover, none of them are widely implemented in industrial applications due to the equipment cost, complex set-ups and long processing times.

For this reason, different techniques to monitor or evaluate tool wear [37,38,39] have been developed, using indirect measurements of the cutting tool wear through monitoring systems [40] or simulations [41], and mainly focused on orthogonal cutting and chip formation [42].

There are different image processing methods applied to the control of tool wear [43], but the most recent method proposed came from Trujillo et al. [27], in whose study the area affected for tool wear is selected manually and measured with specific software. This is a simple technique and industrial implementation is possible at low cost.

In this research paper, the evolution of the adhesion wear mechanism in the dry turning of AA2024 (Al-Cu) is reported. The mechanism induces a positive volume deviation of the cutting tool and uses an automated image processing technique that allows for easy, quick and cheap evaluation.

Turning is a well-known machining process with two characteristics that make it ideal for this study. It is a process close to orthogonal cutting conditions and the cutting tool geometry is simple. It is also widely used in industries where light alloys are considered as strategic materials, such as the aerospace industry, where the application of eco-friendly processes is a challenge nowadays.

## 2. Materials and Methods

Tests were performed in cylindrical samples of aluminium alloy AA2024-T3 (Al-Cu), and carried out in a CNC Lathe EMCOturn 242 (EMCO, Hallein, Austria), with 13 kW power and EMCOtronic TM02 numerical control.

Tests were designed to study the evolution of the wear elements depending on the cutting parameters, using combinations of four different cutting speeds, four different feed rates, and three different depths of cut. The machining time was established in 10 s in all cases and the tests were executed in the absence of cutting fluids (dry machining). Therefore, 48 different tests were carried out. Table 1 collects the different parameters used. The cutting parameters have been selected from those industrially used, according to previous works [27]. The short machining time was considered in order to analyse the first instant of machining [44].

In order to reduce the interference of the cutting tool geometry in the development of the machining tests, a close situation to orthogonal cut is chosen. An uncoated commercial carbide tungsten insert with 10% Co from SECO (ref. ISO DCMT11T308) [45] was chosen. The selection of the uncoated insert is done in order to prevent masked tool wear behaviour by coating. The main cutting insert features are: lip angle 55°, clearance angle 7°, rake angle 17°, and classic chip-breaker geometry for fine cutting application and general purpose.

**Figure 1:**
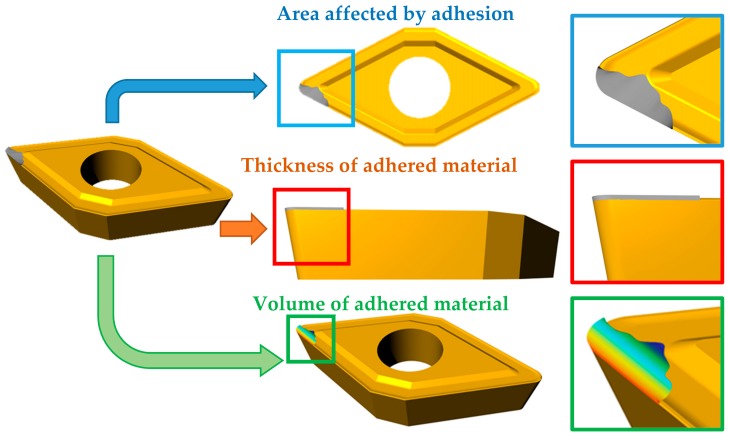
Scheme of the experimental methodology.

The cutting tool characterisation was carried out in three steps (Figure 1). The two first steps of the evaluation were done using a Stereoscopic Optical Microscope (SOM) Nikon SMZ-800 (Tokyo, Japan) connected to a 5 Mpx optical camera Optikam B5 (Optika, Ponteranica, Italy). Additionally, an optic-fibre lighting ring with low reflectance was used as the illumination system. After calibration, a precision of 0.94 µm/pixel was calculated.

**Figure 2:**
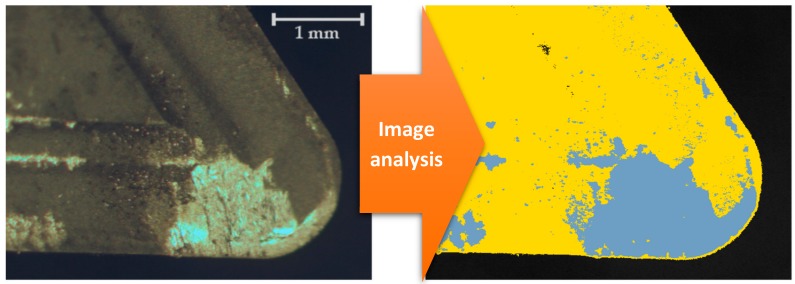
Example of the affected area by adhesion (v_c_ = 192 m/min, f = 0.3 mm/rev, a_p_ = 1 mm, t = 10 s).

The first step is a phase analysis. In this case a Perfect Image v7 (Clara Vision, Verrières le Buisson, France) software was used. The image is filtered and colour patterns are recognised automatically. This technique allows us to establish the extension of the adhered material. It is calculated as a percentage of the affected area on the rake face by the adhesion. This extension is evaluated in terms of percentage occupation from a zenith image processing based on projection. All the images are taken using the same magnification and the same area of the tool is examined. Figure 2 shows an example of the difference of colour corresponding to the adhered material (grey). The same image processing procedure was used for every tool, with three iterations for each image.

**Figure 3:**
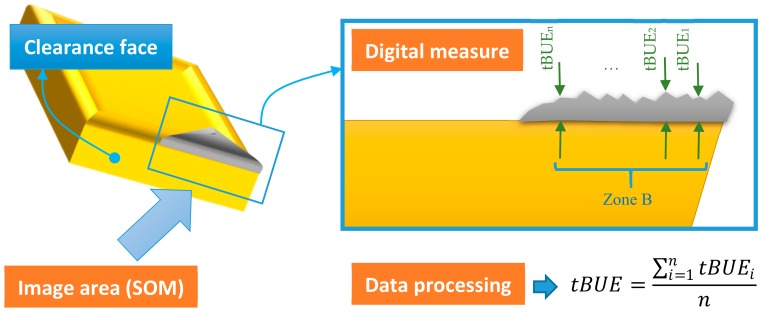
Built-Up Edge (tBUE) thickness measurement.

The second analysis is of the thickness of the material adhered on the cutting edge (BUE). A minimum of 10 equally spaced images was taken in Zone B, according to the actual standard ISO 3685 [46] before calculating the mean value. This measurement process is shown in Figure 3.

The area affected by adhesion and its thickness of cutting tool are two-dimensional parameters that define the volume of material adhered of the cutting tool. In order to establish a three-dimensional relationship with the tool wear, the difference in volume of the cutting tool before and after the cutting process was studied. The technique used to study this variation is contact profilometry, using a profilometer TALYSURF CLI 1000 (Taylor Hobson, Leicester, UK). The profiles defined by the topography generate a three-dimensional image of the material adhered to the tool. Adhered volume can be compared with the topography of a new cutting tool using the software Talymap Platinum v5 (Taylor Hobson, Leicester, UK) in order to obtain the volume of adhered material.

The acquired area was 6.15, 6.25 and 8.75 mm^2^ for 0.5, 1 and 2 mm depth cuts, respectively. Five thousand data points are taken in each profile and the space between them is 0.0005 mm.

A section of the tool cut on the perpendicular plane to the cutting edge is studied using a Scanning Electron Microscope (SEM).

## 3. Results

### 3.1. Evaluation of the Thickness of the Adhered Material onto the Cutting Edge

**Figure 4:**
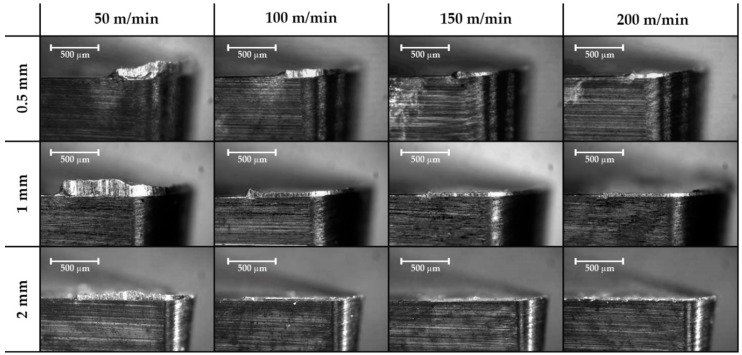
Effect of the feed rate cutting speed and the depth of cut on the secondary adhesion wear in the lateral plane for a 0.2 mm/rev feed rate (63×).

**Figure 5:**
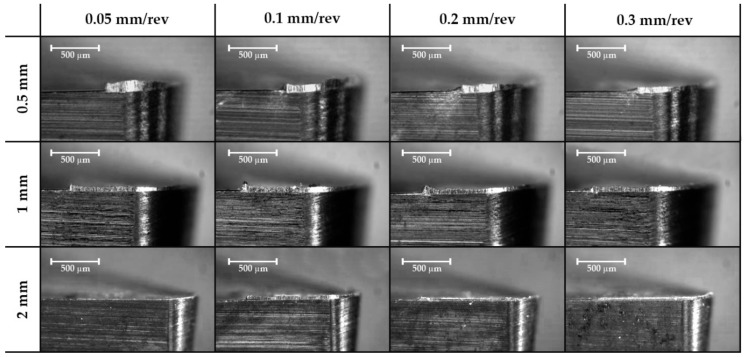
Effect of the feed rate and the depth of cut on the secondary adhesion wear in the cenital plane for cutting speed 100 m/min (63×).

Images were taken perpendicularly to the rake face in order to obtain the real thickness of the material adhered to the cutting edge. This adhered material is considered the Built-Up Edge or BUE. Marks of chip fluency are also observed in the clearance face but disappear with a high depth of cut, so may be related to the cross-sectional area of the chip. It can be appreciated that a lower cutting speed is more suitable for presenting a Built-Up Edge (Figure 4), while an increase in the feed rate can reduce this effect (Figure 5).

**Figure 6:**
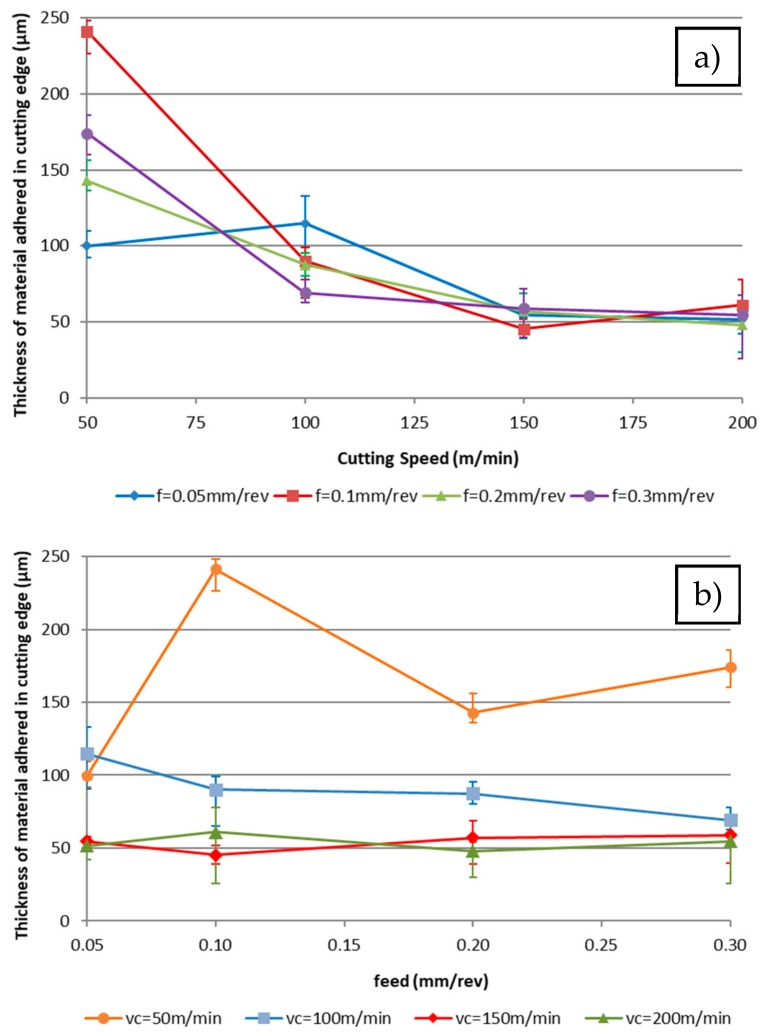
Effect of the thickness of the material adhered on to the tool edge on the secondary adhesion wear for 0.5 mm depth of cut depending on the (**a**) cutting speed; (**b**) feed rate.

Numerical results show a similar trend to the visual ones. Figure 6 represents the average thickness of the adhered material depending on the cutting speed and the feed rate for a 0.5 mm depth of cut. It has been verified that this behaviour is similar for the other depths of cut.

The highest values of adhered material are observed for lower cutting speeds, having a similar behaviour for all depths of cut. When the depth decreases, the thickness of the material adhered to the tool is reduced. There is also a decreasing trend related to the cutting speed that plateaus in a horizontal line.

Therefore, the trends are affected by the depth of cut. A lower depth of cut increases the thickness of the adhering material. The slope of this trend decreases when the depth of the cut increases. This effect can be related to the chip forming process. In this case, for the highest feed rates, the cross section of the chip is higher and the associated chip tension is higher too. Due to this, the chip form is long or snarled, in agreement with Rubio et al. [47].

Also, the thickness of the adhered material decreases slightly with the feed rate, while an increase in the cutting speed reduces the sensibility of the feed change. For a high cutting speed, the slope of this trend is higher. This behaviour is related to the temperature of the process, which promotes the mechanical adhesion [48].

Moreover, for extreme values of cutting speed and feed rates, there are higher differences due to the increase of cutting forces. Chip width sense is smaller when decreasing those forces, considering the chip projection over the cutting edge. This behaviour was observed in the cutting force by Carrilero et al. [49].

The experimental results suggest that it is possible to obtain a potential parametric model of X = f(v_c_, f, a_p_) with a better fit than other models. This was in accordance with the statistical treatment proposed for similar studies [27,50,51].

Potential models have been proposed for each depth of cut:

(1) $$\;\mathrm{tBUE}\;\left(a_{p}=0.5\;\mathrm{mm}\;\right)=3985.58{\cdot f}^{-0.0002}{\cdot v}_{c}{}^{-0.832}$$

(2) $$\;\mathrm{tBUE}\;\left(a_{p}=1\;\mathrm{mm}\;\right)=1002.24{\cdot f}^{0.356}{\cdot v}_{c}{}^{-0.461}$$

(3) $$\;\mathrm{tBUE}\;\left(a_{p}=2\;\mathrm{mm}\;\right)=384.76{\cdot f}^{0.327}{\cdot v}_{c}{}^{-0.432}$$

Those models present the little variations analysed before as well as the high variability of the results. A combined model has been developed from the previous models:

(4) $$\;\mathrm{tBUE}\;=1244.205{\cdot f\;}^{0.266}{\cdot v}_{c}{}^{-0.567}{\cdot a}_{p}{}^{-0.806}$$

**Figure 7:**
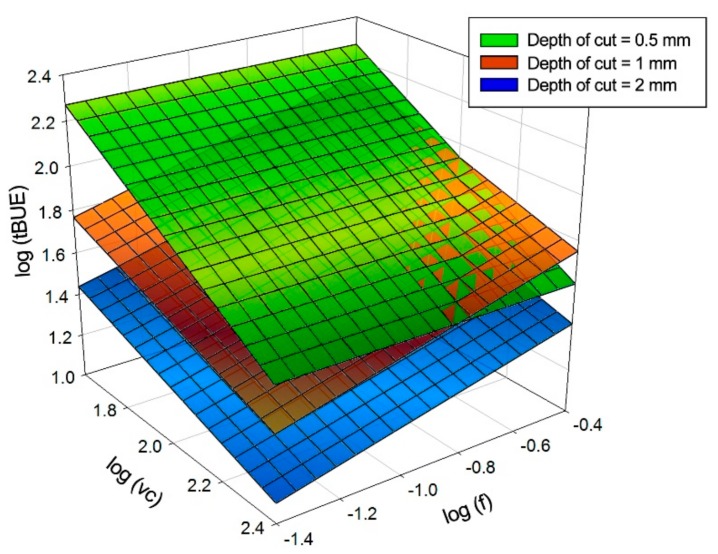
Graphic representations of the models calculated for the thickness of the material adhered on the cutting edge.

Figure 7 presents three-dimensional models and the combination of them, showing the trends previously mentioned.

**Figure 8:**
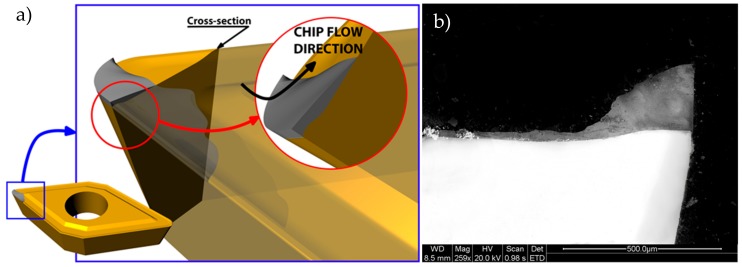
(**a**) Scheme of the cross section of the tool; (**b**) SEM image of this cross section.

It is remarkable that the material adhered on the cutting edge (BUE) changes the geometry of the cutting tool. This change modifies the clearance angle and the rake angle. An example of the new geometry is seen in the transversal section of the tool (Figure 8). In this case, the chip flows out of the rake face and the contact pair in this cutting condition is aluminium–aluminium. This affects the friction coefficient and the temperature of the process, while the contact area decreases [17]. Also, as is shown in Figure 8, the thickness of the adhered material on the cutting edge is higher than that of the material adhered on the rake face.

### 3.2. Evaluation of the Area Affected by Adhesion

**Figure 9:**
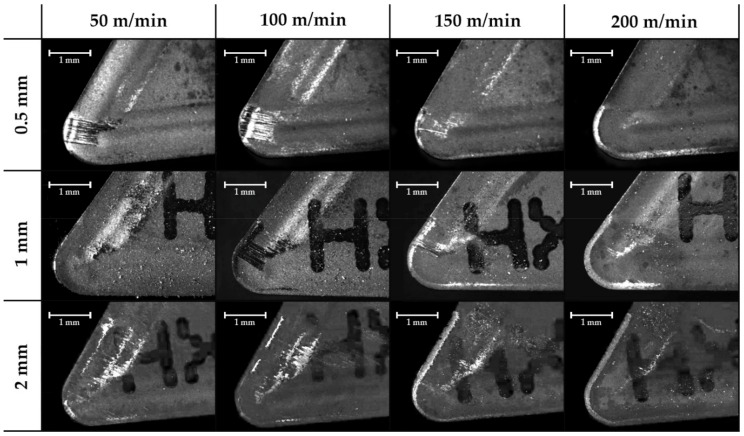
Effect of the cutting speed and the depth of cut on the secondary adhesion wear in the zenith plane for 0.05 mm/rev feed rate (30×).

**Figure 10:**
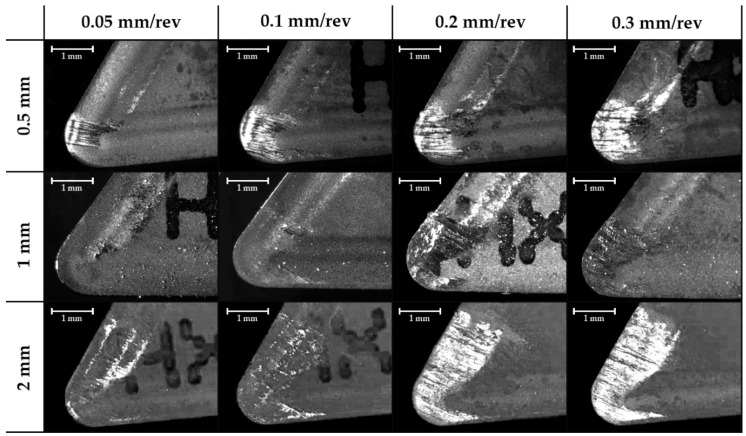
Effect of the feed rate and the depth of cut on the secondary adhesion wear area in the zenith plane for cutting speed 50 m/min (30×).

The images taken in the position perpendicular to the rake face show a layer adhered on this face, considered the Built-Up Layer or BUL. Also, it is possible to see a decrease in the affected area by adhesion when the cutting speed increases (Figure 9). Additionally, there is an increase in the affected area related to the increase of the feed rate (Figure 10). This behaviour is independent of the depth of cut.

**Figure 11:**
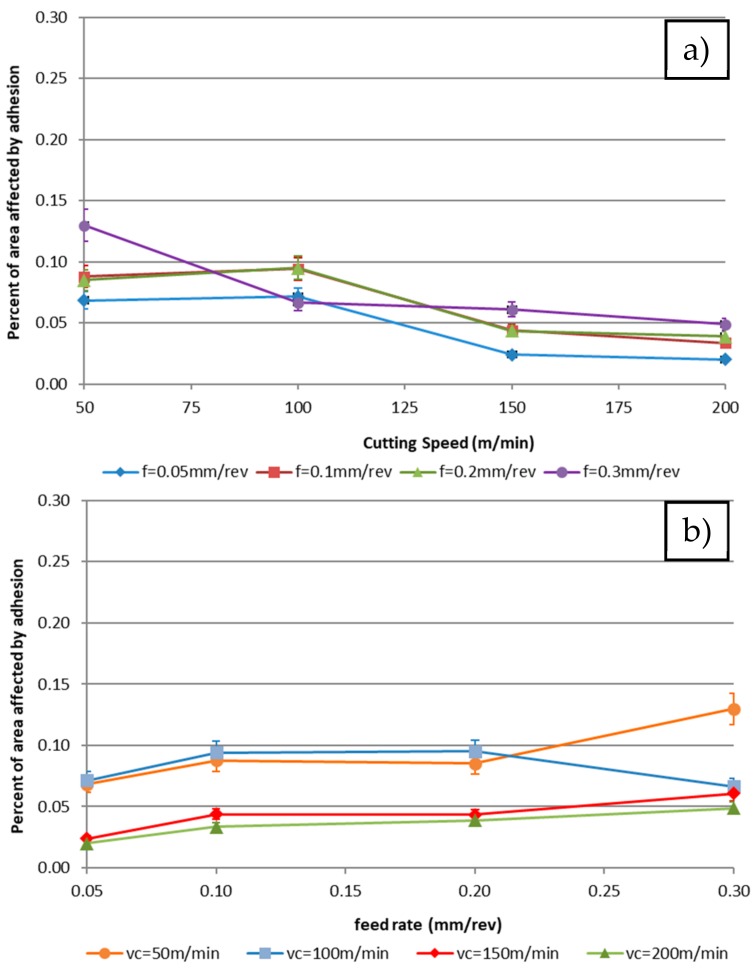
Effect on the area affected by secondary adhesion (%A) for 0.5 mm depth of cut depending on the: (**a**) cutting speed; (**b**) feed rate. Data measured by image processing techniques.

Figure 11 shows the evolution of the % area affected by adhesion wear, according to the cutting speed and the feed rate using 0.5 mm depth of cut.

Analytical data show the same trends observed in the images. There is a decreasing trend of adhesion area when the cutting speed increases for all the studied feed rates. These trends seem to be constant and unaffected by the feed rate.

This fact is related to the cutting temperature increment that appears with the rise in the speed, as was analysed by [44,52]. Moreover, a horizontal trend is noted for the highest cutting speed values, which can be related to a stabilisation of this parameter. This result matches the results obtained in the test performed by [29] in a high-speed machining test.

In this case, the reduction of the affected area can be related to the tool geometrical change explained before. An increase in the cutting edge adhesion induces a rise in the contact length between the chip and the tool, reducing the adhesion effect. This phenomenon has been verified in orthogonal cut by Atlati et al. [53]. This effect is related to the resilience of the material, obtaining opposite tendencies to those observed in Trujillo et al.’s [27] experiments. Those differences are due to the use of aluminium with the highest resilience in Trujillo’s tests. Also, it has to be considered that the method used by Trujillo et al. is manual and an automatic one can be more robust.

**Figure 12:**
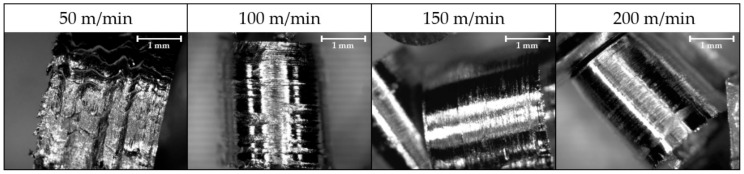
Chip contact face for different cutting speed at 0.2 mm/rev feed rate and 2 mm depth of cut (30×).

**Figure 13:**
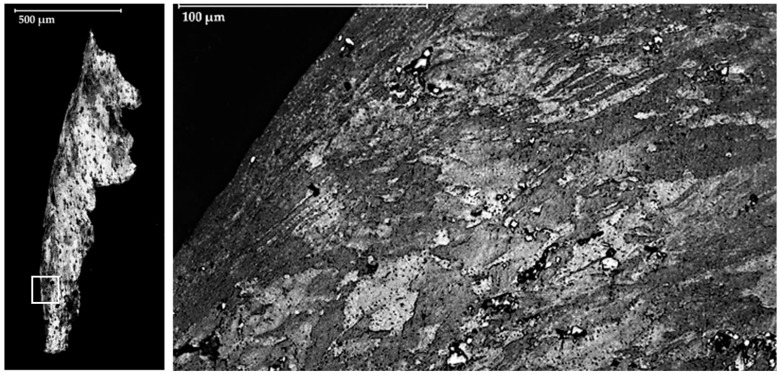
Metallographic optical microscopy of the chip cross section.

The surface of the chip varies due to the lack of contact between tool and material. For the highest cutting speed, the number of scratches, marks and defects on the chip surface are reduced (Figure 12). Those defects are caused by the type of flow over the rake face. It can be noted that on the contact area the grains are elongated in the longitudinal sense (Figure 13).

We expected a higher affected area because of the increase in the depth of cut caused by the increase of the contact area between the part and the tool. For this reason, for a smaller depth of cut, the area affected is smaller. However, the parametric trends seem to be constant for every depth of cut studied.

Despite the fact that adhesion is a cyclical process and the adhered material is removed and reformed in the evaluated period [18], the deviation of the data is not remarkable. This phenomenon could hide other effects of adhesion on the tool rake face, like a possible crater. For this reason, this method does not draw any distinction between primary and secondary BUL, which limits the evaluation to the material extension.

From the obtained data, several marginal models of the depth of cut have been calculated. The objective is to make the graphic representation of the joining model easier:

(5) $$\;\%A\;\left(a_{p}=0.5\;\mathrm{mm}\;\right)=359.317{\cdot f}^{0.301}{\cdot v}_{c}{}^{-0.752}$$

(6) $$\;\%A\;\left(a_{p}=1\;\mathrm{mm}\;\right)=120.462{\cdot f}^{0.529}{\cdot v}_{c}{}^{-0.317}$$

(7) $$\;\%A\;\left(a_{p}=2\;\mathrm{mm}\;\right)=57.968{\cdot f}^{0.850}{\cdot v}_{c}{}^{-0.006}$$

A combined model based on the overlap of these models is established:

(8) $$\;\%A=146.657{\cdot f\;}^{0.551}{\cdot v}_{c}{}^{-0.371}{\cdot a}_{p}{}^{0.421}$$

**Figure 14:**
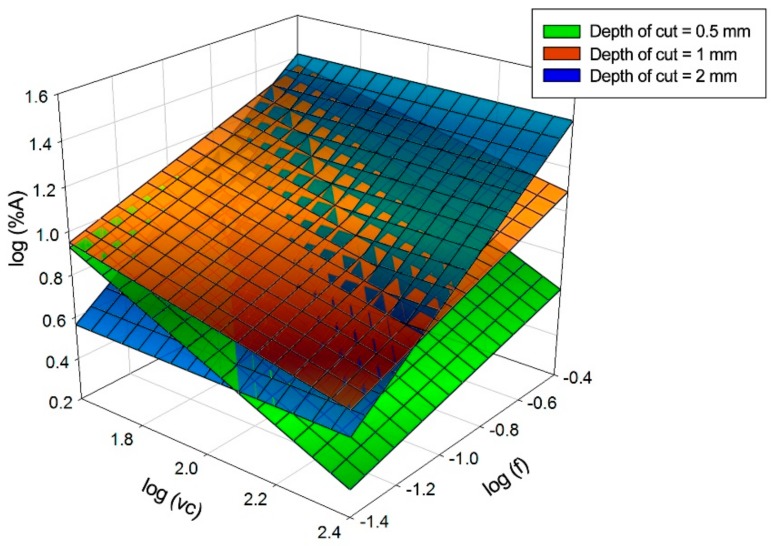
Graphic representations of the models calculated for the secondary adhesion-affected area.

It is observed that the exponent of the cutting speed points, a horizontal trend for the highest values of the depth of cut and the increase of depth of cut is shown as a high exponent of the feed rate. Figure 14 presents the superposition model. This figure reflects the trends of behaviour explained previously. The planes are cut because the trends are inverted when the depth of cut increases.

As presented in the model, the sensitivity of the geometry change depends on the depth of cut. With a higher value of this parameter, the tool is more sensitive to changes caused by an increase in the feed rate and less so for those caused by an increase in the cutting speed, due to an increase of the chip section and an increase in the specific cutting tension. This is related with a highest stress to perfume the cutting process, which is also linked to an increase in the temperature in the cutting zone.

### 3.3. Volume of the Material Adhered of the Cutting Tool

**Figure 15:**
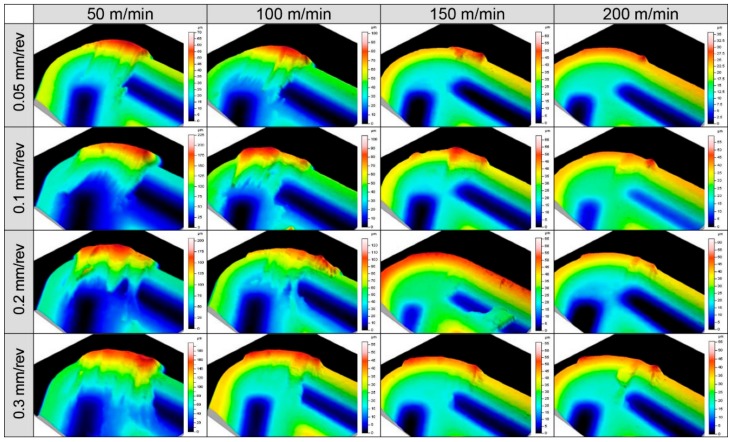
Comparative topography of the tools used in 0.5 mm depth of cut machining tests.

In this case, similar to in a previous analysis, we studied the tools obtained for each depth of cut. Figure 15 shows the topographies obtained for a 1 mm depth of cut. However, the same trends were found for the other depths of cut.

This is in accordance with what was observed in previous cases and can be explained by the tension of the chip and the lack of contact with the tool.

**Figure 16:**
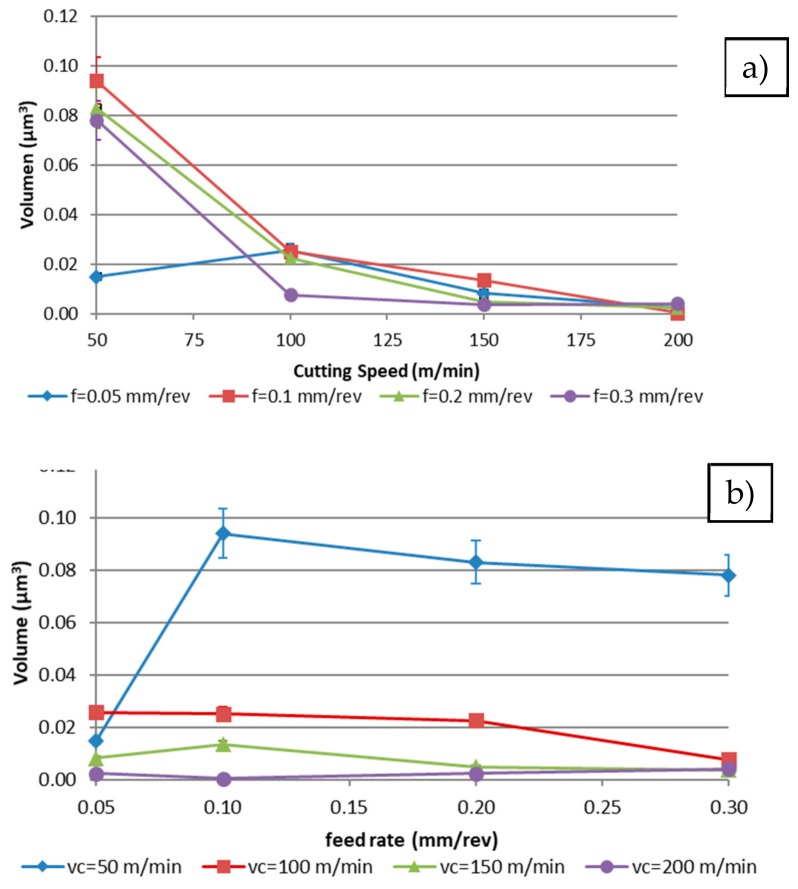
Effect of the (**a**) cutting speed; (**b**) feed rate on the volume of the material adhered to the cutting tool (V) for 0.5 mm depth of cut.

Figure 16 shows the effect of the cutting parameters on the volume of the material adhered to the cutting tool for 0.5 mm depth of cut. Others depths of cut shows the same trends.

**Figure 17:**
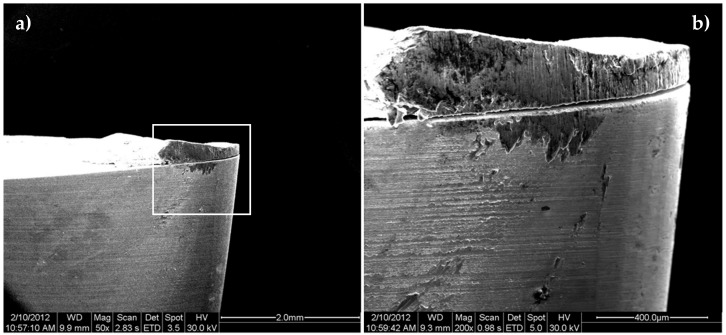
SEM of a tool in the previous instants to the detachment of the adhering layer. (**a**) 50× magnification; (**b**) 200× magnification.

It is difficult to establish homogeneity given the dynamic behaviour of the adhesion mechanism. As shown in Figure 17, the adhering material can be easily removed (the whole adhesion layer or a part of it). This continuously alters the behaviour of the tool, changing the performance of the process.

According to the data obtained, a potential model has been developed as shown in Equation (9):

(9) $$\;V\;=2.121{\cdot f\;}^{1.463}{\cdot v}_{c}{}^{0.501}{\cdot a}_{p}{}^{0.111}$$

**Figure 18:**
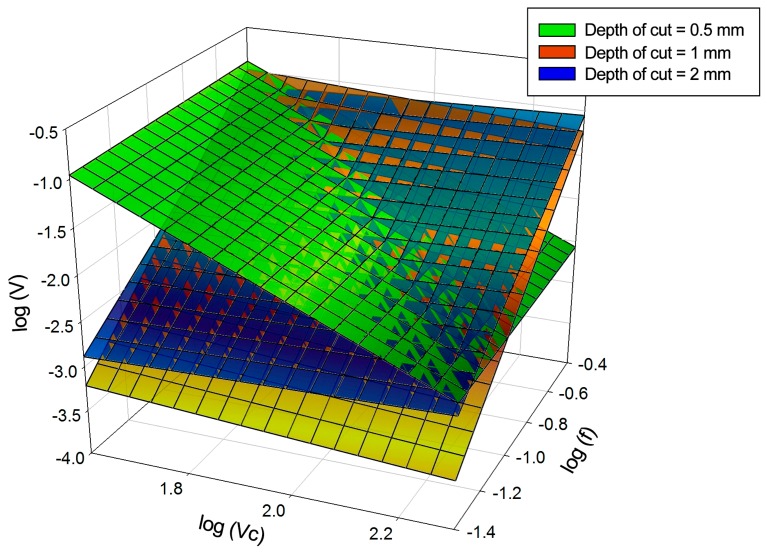
Graphic representations of the models calculated for the volume of adhering material.

Figure 18 shows the regression planes of the marginal models and the joint model. The model represents the union of the two effects already studied. It is also noted that the most material is adhered to the cutting edge, while the material adhered on the rake face has a lower thickness and volume.

## 4. Conclusions

This paper has evaluated the secondary adhesion wear in dry turning of an AA2024 aluminium alloy using a specific methodology. The three effects of the secondary adhesion wear were studied: the affected area in the rake face (BUL), the thickness of material adhered over the cutting edge (BUE), and the total volume adhered on the tool (BUL/BUE). Those parameters are studied under two-dimensional optical techniques and with a 3D profilometer. Parametric models for the three effects are proposed by combining different cutting parameters.

The thickness of material adhered on the cutting edge shows more dependence on the cutting speed than the feed rate. A layer of higher thickness appears with a lower cutting speed. Similar trends are noted with depth of cut. The geometry of this adhering material also changes the geometry of the tool; this change does not have linear behaviour.

The affected area in the rake face is influenced by this geometrical change. For this reason, when the thickness of the material adhered on the cutting edge is increased, the contact length of the chip–tool pair is lower and the affected area of the rake face is decreased. Thus, the influence of the cutting speed is lower and the feed rate is higher. The surface of the chip shows this behaviour with a clear surface.

The adhesion wear is a dynamic mechanism. For this reason, there is instability in the wear mechanism. This instability is observed in some tools when the material adhered can be removed easily. This may affect the instantaneous results, but the general trend is not affected.

However, the study of the volume of the material adhered to the tool shows a higher dependence on the material adhered in the cutting edge because it presents a lower thickness to a rake face. In this way, the volume adhered on the tool shows a similar trend to the thickness of the material adhered on the cutting edge.

This study uses two different techniques, a basic optical technique and a complex metrological technique. The optical technique allows for the measurement of the tool wear with high precision, making it a suitable technique for industrial implementation due its simplicity, low cost and precision. This study also sets up a foundation to establish an automatic calibration system for a tool based on optical techniques. This can minimise the negative effects of secondary adhesion wear.

## Figures and Tables

**Table 1:** Cutting parameters.

Cutting Speed (v_c_) (m/min)	50	100	150	200
Feed (f) (mm/rev)	0.05	0.1	0.2	0.3
Depth of cut (a_p_) (mm)	0.5	1	2	-
Time of cut (t) (s)	10			
